# Protocol for safe, affordable, and reproducible isolation and quantitation of SARS-CoV-2 RNA from wastewater

**DOI:** 10.1371/journal.pone.0257454

**Published:** 2021-09-23

**Authors:** Monica Trujillo, Kristen Cheung, Anna Gao, Irene Hoxie, Sherin Kannoly, Nanami Kubota, Kaung Myat San, Davida S. Smyth, John J. Dennehy

**Affiliations:** 1 Department of Biology, Queensborough Community College, The City University of New York, New York City, New York, United States of America; 2 Biology Department, Queens College, The City University of New York, New York City, New York, United States of America; 3 Biology Doctoral Program, The Graduate Center, The City University of New York, New York City, New York, United States of America; 4 Department of Natural Sciences and Mathematics, Eugene Lang College of Liberal Arts at The New School, New York City, New York, United States of America; University of Helsinki: Helsingin Yliopisto, FINLAND

## Abstract

The following protocol describes our workflow for processing wastewater with the goal of detecting the genetic signal of SARS-CoV-2. The steps include pasteurization, virus concentration, RNA extraction, and quantification by RT-qPCR. We include auxiliary steps that provide new users with tools and strategies that will help troubleshoot key steps in the process. This protocol is one of the safest, cheapest, and most reproducible approaches for the detection of SARS-CoV-2 RNA in wastewater. Owing to a pasteurization step, it is safe for use in a BSL2 facility. In addition to making the protocol safe for the personnel involved, pasteurization had the added benefit of increasing the SARS-CoV-2 genetic signal. Furthermore, the RNA obtained using this protocol can be sequenced using both Sanger and Illumina sequencing technologies. The protocol was adopted by the New York City Department of Environmental Protection in August 2020 to monitor SARS-CoV-2 prevalence in wastewater in all five boroughs of the city. In the future, this protocol could be used to detect a variety of other clinically relevant viruses in wastewater and serve as a foundation of a wastewater surveillance strategy for monitoring community spread of known and emerging viral pathogens.

## Introduction

Tracking SARS-CoV-2 infections often involves detecting SARS-CoV-2 RNA via RT-qPCR in biological samples obtained from patients that develop symptoms associated with COVID-19 [1]. However, if only patients who seek medical care are sampled, community transmission may be underestimated due to asymptomatic patients or those with mild symptoms who follow the CDC’s advice and convalesce at home [2, 3]. Additionally, SARS-CoV-2 sequencing efforts, while occurring at a much faster rate and larger, more global scale than in previous pandemics, suffer biases because genomic information is often obtained from seriously ill patients, but not from patients who do not seek medical attention. If a significant proportion of cases are asymptomatic or unsampled, SARS-CoV-2 population genetic diversity within communities may be underestimated. Moreover, the surge of novel variants of concern in different regions of the world has added another challenge [4, 5], which is to monitor the proportion of individuals that carry a particular variant in a geographical area. Given that SARS-CoV-2 has been detected in fecal samples [6, 7], and subsequently in wastewater [3, 8, 9], wastewater is being tested in cities around the world to determine SARS-CoV-2 prevalence in communities [10–12]. Furthermore, isolation of SARS-CoV-2 RNA from wastewater coupled with high-throughput deep sequencing provides an almost unlimited source of unbiased viral sequences, which can be used to monitor frequencies of variants of concern in populations.

With the goal of sequencing SARS-CoV-2 RNA from wastewater, we developed a protocol to extract and quantify viral RNA. The initial step in the development of this protocol was the decision to pasteurize our samples at 60°C for an hour on arrival at the laboratory. Given that SARS-CoV-2 is a risk group 3 (RG3) agent, inactivation of the virus before processing is often required before samples can be processed in a BSL2 laboratory. Happily, as we report here, pasteurization did not impair our ability to detect SARS-CoV-2, but instead, improved it. Our protocol includes the spiking-in with a control virus to determine the efficiency of recovery. Interestingly, while SARS-CoV-2 recovery was not impaired by pasteurization, the two control spike-in viruses tested bovine coronavirus (BCoV) [13] and bacteriophage Phi6 [14] were rendered barely detectable using RT-qPCR and PCR respectively by the pasteurization step. Subsequently, control viruses were spiked-in after pasteurization. Furthermore, we noticed no appreciable difference in sequencing quality between pasteurized and unpasteurized samples.

A second major decision was to employ centrifugation and filtering (0.2 μM) to remove wastewater solids which we made at the beginning of our study. While SARS-CoV-2 may associate with solids, removing solids facilitates the downstream processing steps and may remove genomic contamination that would impair our ability to deep sequence SARS-CoV-2 so we adopted filtering early on the development of our protocol. As a counterpoint, filtration is one of the more expensive steps of the protocol so those desiring to reduce costs may consider eliminating filtration. We were not able to acquire consistent results without filtration so we opted to filter all samples following pasteurization.

Since viruses are greatly diluted in wastewater, virion concentration is a significant challenge. We considered three common protocols to concentrate SARS-CoV-2 virus present in the water: ultracentrifugation [15], skimmed milk flocculation [16], and polyethylene glycol (PEG)/sodium chloride (NaCl) precipitation. High speed centrifugation was ruled out as impractical for the volumes needing to be processed. Precipitation/flocculation using PEG/NaCl or skimmed milk eliminates the need for high-speed ultracentrifugation and generates sufficient RNA for viral quantification with RT-qPCR (i.e., resulting in Cts < 40). However, in our experiments, PEG/NaCl precipitation performed marginally better than skim milk flocculation and did not introduce additional genetic material to our samples so this was chosen as our concentration method. Additionally, we explored the effect of longer incubation times on viral RNA recovery. Longer storage in PEG/NaCl of the pasteurized samples led to slightly better virus recovery, but the difference was not significant.

As we were mindful of the need to find cost effective solutions, we investigated alternative, kit-free approaches to RNA isolation. In our hands, TRIzol (ThermoFisher Inc.) performed better than the QIAamp Viral RNA Mini kit (Qiagen Inc.). As TRIzol is cheaper per sample than column-based kits, we adopted it for the final protocol. An added benefit of TRIzol relevant to downstream sequencing applications is that TRIzol segregates RNA in a separate layer from DNA, unlike column-based isolation kits, which isolate both RNA and DNA.

In addition to the RNA isolation method, we compared the performance of different RT-qPCR enzymes, TaqPath 1-Step RT-qPCR enzyme (Thermofisher Inc.) and One Step PrimeScript III enzyme (Takara Bio USA Inc. The RT enzyme from Takara had a similar performance to Taq-Path so we chose it for the final protocol. A broader investigation of different enzymes may identify other satisfactory, cost-effective solutions.

Our protocol provides a reproducible and low-tech approach that allows the detection and quantification of SARS-CoV-2. Pasteurization of the sample at the very beginning of the protocol ensures the safety of the user. Preliminary results suggest that pasteurization may also release the virus bound to the wastewater solids, enhancing recovery. Filtering and PEG/NaCl concentration simplifies downstream processing. We were able to perform both targeted and whole genome sequencing of the SARS-CoV-2 genome using this protocol.

Our protocol performed strongly in a large-scale, nationwide comparative study of the reproducibility and sensitivity of 36 methods of quantifying SARS-CoV-2 in wastewater [17]. Our protocol is identified as 4S.1(H) in the Pecson et al. study [17]. In addition, the Pecson et al. study offers strong support for several of the primary claims of the present paper. First, the removal or non-removal of the wastewater solids did not show a clear systematic impact on outcomes. Second, pasteurization resulted in a small, but significant, increase in recovery. Third, methodological differences between teams had minimal impact on reproducibility and sensitivity, thus indicating that our modifications to implement cheaper, simpler methods will not impair SARS-2-CoV-2 detection and quantification relative to other strategies.

We recognize that our protocol has some limitations. Our current protocol isolates the RNA from 40 ml of wastewater and requires access to a centrifuge capable of reaching 12,000 x g. Thus, scaling up the volume of samples from 40 ml or increasing the number of individual samples, represents a challenge. Our protocol requires filtration units which are dependent on the supply chain. Additionally, extracting RNA with TRIzol requires the user to take care not to contaminate the aqueous phase with organic material after centrifugation, which can be difficult for inexperienced users. Nevertheless, the basic protocol and techniques involved are economical, simple, and reproducible when compared to alternative strategies.

## Materials and methods

*The protocol described in this article is published on**protocols*.*io*, *https*:*//www*.*protocols*.*io/view/protocol-for-safe-affordable-and-reproducible-isol-bwvmpe46**and is included for printing as S1 File.*

## Expected results

**Fig 1:**
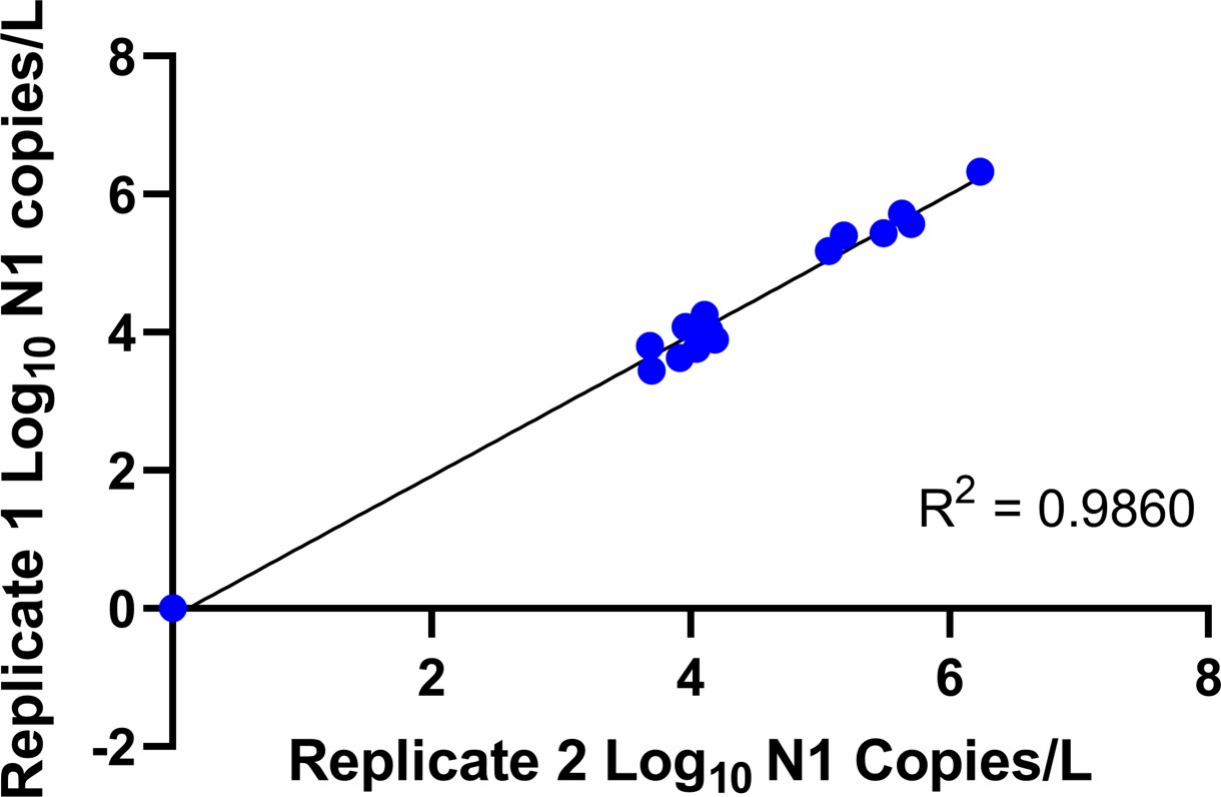
Repeatability of protocol: Pearson correlation of replicate measurements (n = 2) of copy number yield for the N1 target from fourteen 24-hr composite wastewater samples and a negative control, demonstrating the reproducibility of our protocol. Sample collection and initial processing was performed on the same day.

Our protocol results in the reproducible isolation and quantification of SARS-CoV-2 RNA from wastewater samples (Fig 1; Pearson correlation: r^2^ = 0.9860, p < 0.0001). Enough RNA can be acquired for RT-qPCR and isolated RNA is suitable for whole genome amplification and sequencing. As a general note, wastewater treatment plants indicated in our figures have been deidentified. There is no correspondence between the numerical wastewater treatment plant (WWTP) IDs in different figures. Moreover, experiments described in different figures were performed at different times using different wastewater samples. Our purpose here is not to report regional prevalence, but rather to demonstrate the reliability and consistency of our protocol.

Key steps were optimized during the development phase of our protocol. Reports from the scientific community suggested that BCoV would serve as a good control, and because of degradation by pasteurization, we switched to spiking samples with BCoV after pasteurization and before the first centrifugation to remove solids. It would be interesting to determine why BCoV was rapidly degraded by pasteurization, but an ostensibly similar virus, SARS-CoV-2, was not.

**Fig 2:**
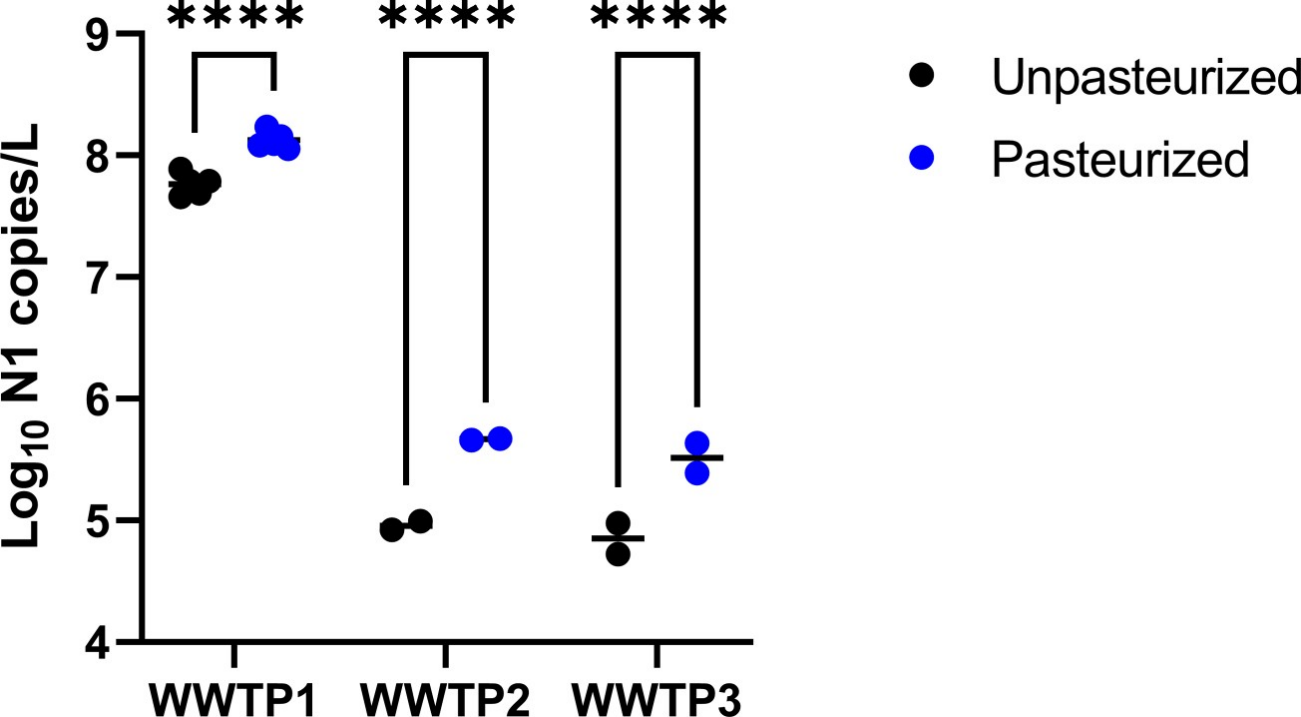
Effect of pasteurization: Copy number yield for the N1 target obtained from three 24-hr composite wastewater samples processed either with or without pasteurization (n = 5 for WWTP1 and 2 for WWTP 2 & 3). Each point is the mean of three technical replicate measurements. Horizontal black lines show means and asterisks show significance level in pairwise comparisons where ns = P > 0.05; * = P ≤ 0.05; ** = P ≤ 0.01; *** = P ≤ 0.001; **** = P ≤ 0.0001.

To ascertain the impact of pasteurization on SARS-CoV-2 quantitation, wastewater samples from three separate WWTPs were divided in half and processed either with pasteurization or without. More SARS-CoV-2 N1 copies/L were detected in the pasteurized samples than in the unpasteurized samples demonstrating the positive impact of pasteurization on SARS-CoV-2 quantification (Fig 2; 2-way ANOVA with Bonferroni correction: F = 67.86, df = 1, p < 0.0001). We speculate that incubation of samples at 60°C contributes to release of virus from wastewater solids. As an additional advantage, pasteurization appears to increase repeatability of sample quantification. The standard deviations for pasteurized and unpasteurized samples were 0.07 and 0.09 respectively. Samples 2 and 11, the most variable sites, are from plants with a significant influx of ocean water, but it is not clear if this is driving variation in these sites. We conclude that pasteurization results in greater sensitivity and more precise estimates of SARS-CoV-2 prevalence. We did similar pasteurization experiments with wastewater samples from additional NY plants and observed the same trend. Similar results have been reported in other studies [17].

**Fig 3:**
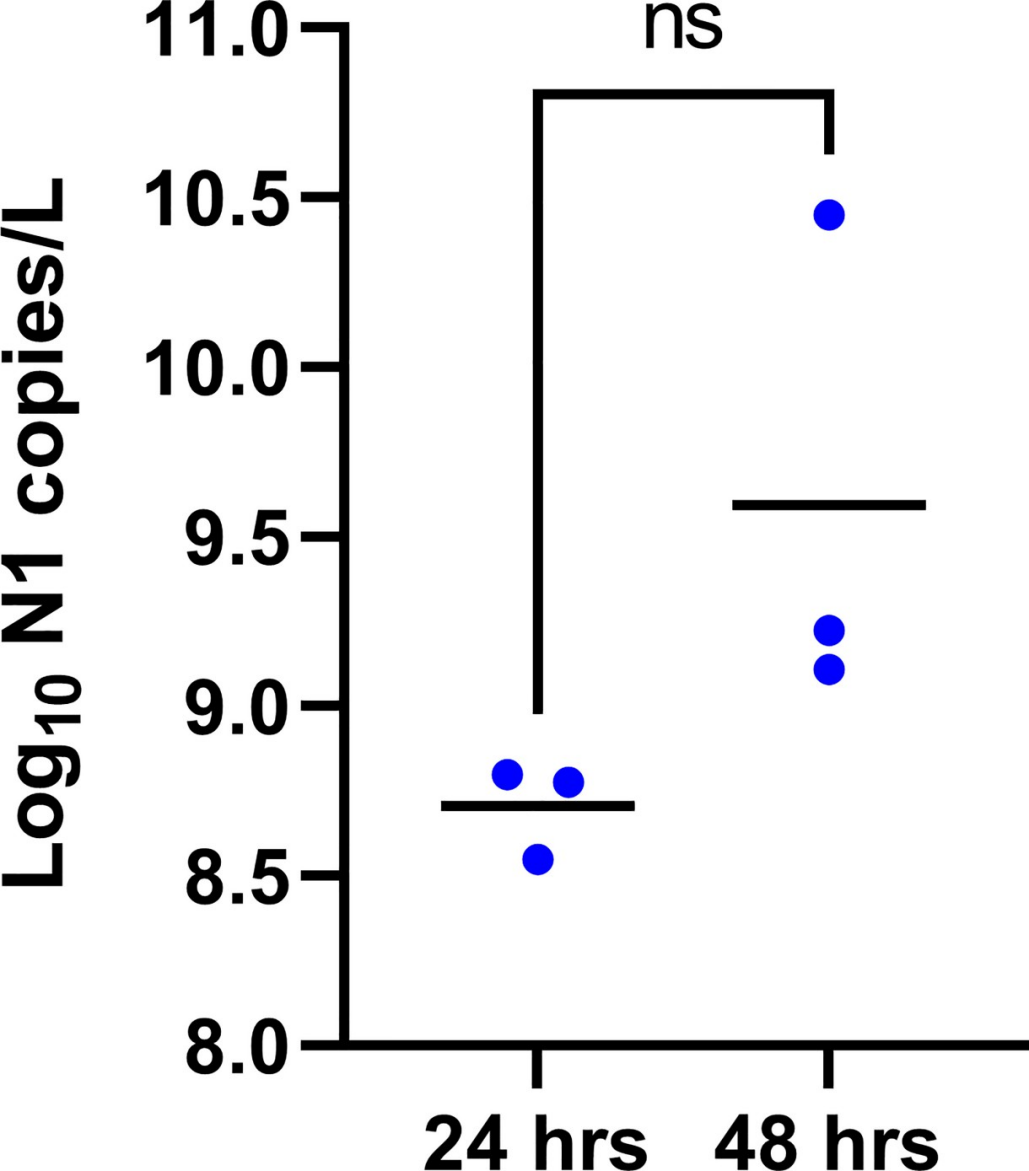
Effect of storage at 4°C in PEG/NaCl: Following initial processing (pasteurization, preliminary centrifugation, and filtering), 24-hr composite samples from 3 different wastewater treatment plants were stored at 4°C in a PEG/NaCl solution for precipitation and concentration of virions. Each point is the mean of two technical replicate measurements from a 24-hour composite sample. Horizontal black lines show means. See Fig 2 legend for explanation of pairwise comparison.

**Fig 4:**
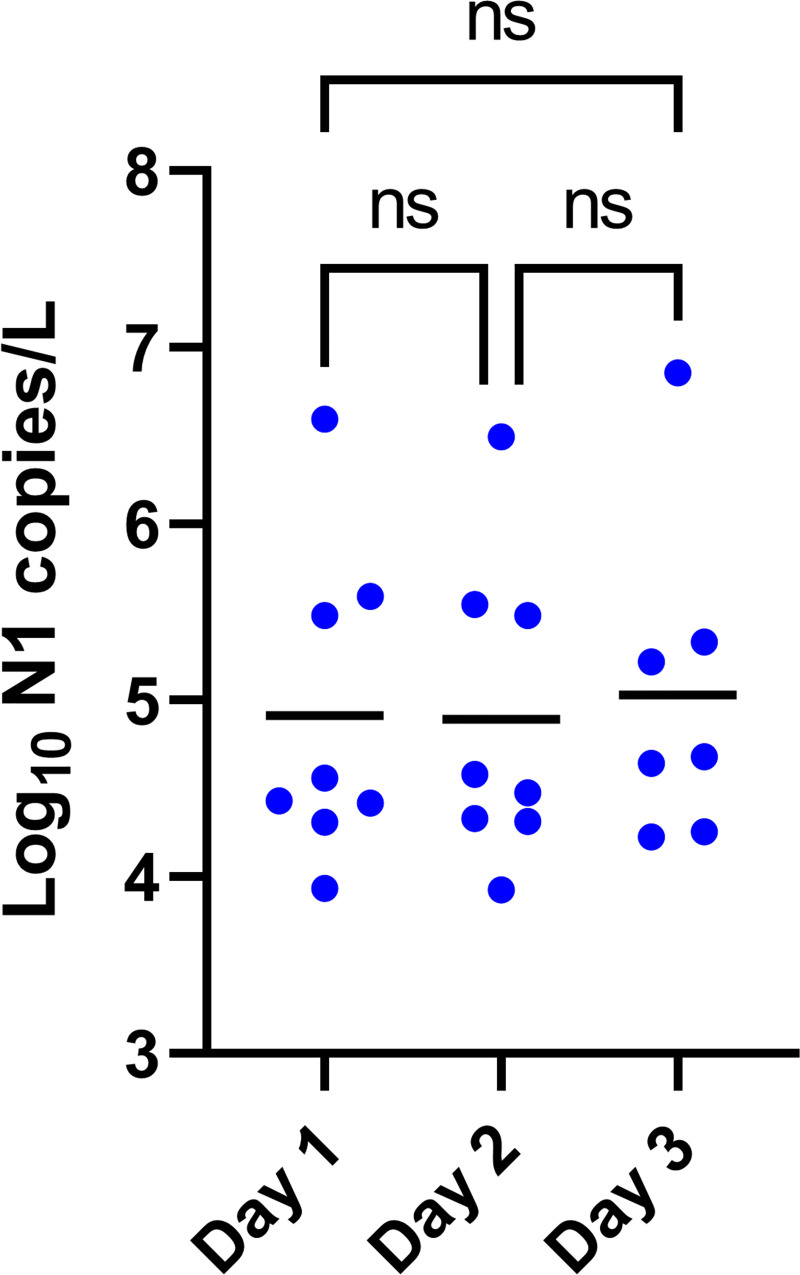
Effect of storage at 4°C on unpasteurized samples: Following initial processing (pasteurization, preliminary centrifugation, and filtering), 24-hr composite samples (n = 8) were stored at 4°C for 72 hrs. Each point is the mean of two technical replicate measurements from a 24-hour composite sample. Horizontal black lines show means. See Fig 2 legend for explanation of pairwise comparisons.

In previous work on bacteriophages, longer PEG/NaCl incubation increased phage recovery (JJD, personal observation). To determine if longer incubation similarly impacts SARS-CoV-2 recovery, we compared SARS-CoV-2 quantitation for samples incubated in PEG/NaCl for 24 hrs versus 48 hrs. While longer storage resulted in slightly improved virus recovery, the difference was not significant (Fig 3; paired t-test: t = 1.745, df = 2, p = ns), an outcome also observed in other studies [18, 19]. In addition, we tested the effect of storage of unpasteurized samples at 4°C without added PEG/NaCl on virus recovery. Storage of pasteurized samples without PEG/NaCl for 72 hrs had no effect on virus recovery (Fig 4; mixed-effect analysis with Bonferroni correction: F = 0.05, p = ns). These results should ameliorate concerns about longer term storage of wastewater samples if they cannot be processed immediately.

**Fig 5:**
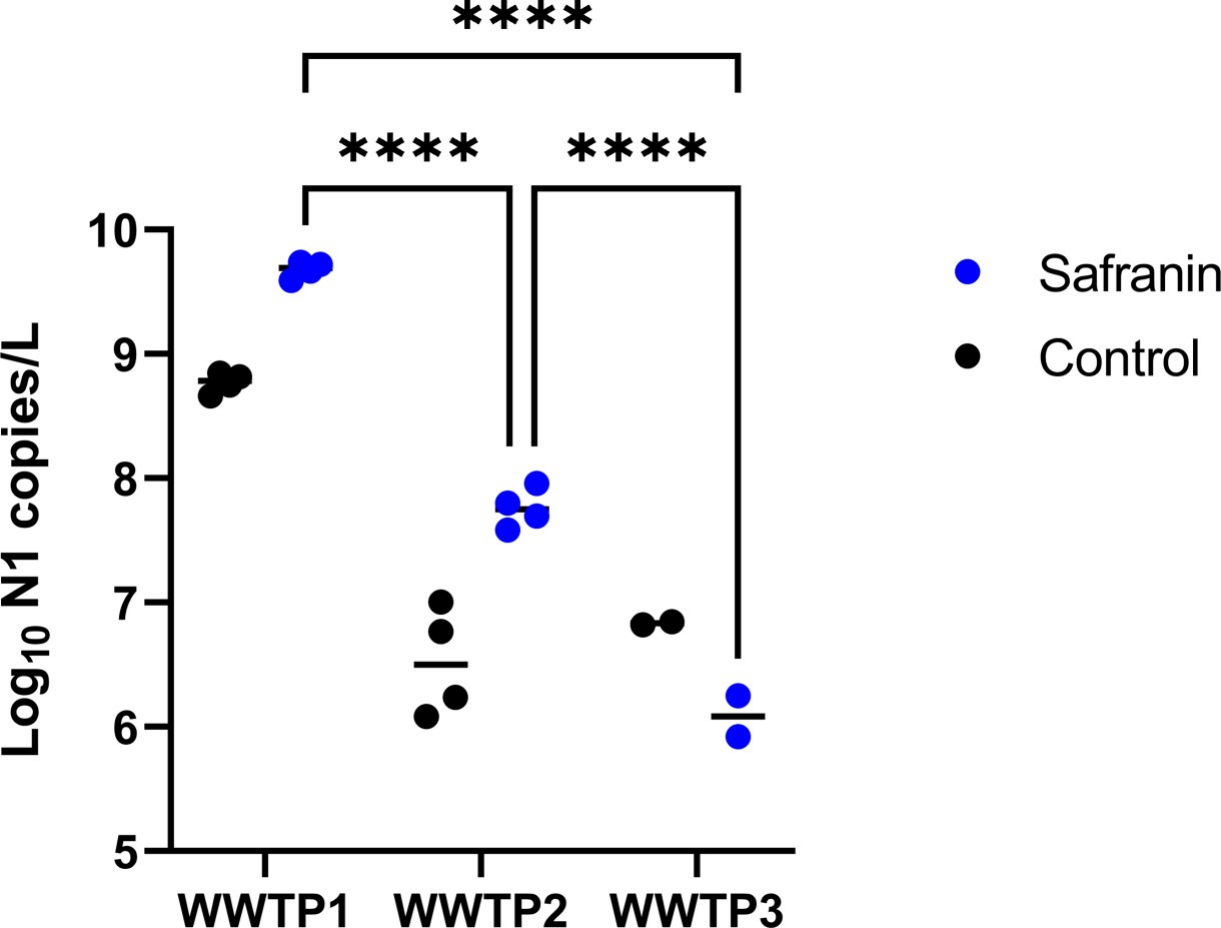
Effect of safranin staining: Copy number yield for the N1 target for 24-hr composite wastewater samples obtained from 3 wastewater treatment plants. Samples processed with safranin are labeled by dark blue circles; controls are labeled with light blue circles. Points are technical replicate measurements from a 24-hour composite sample. Horizontal black lines show means. See Fig 2 legend for explanation of pairwise comparisons.

After PEG precipitation and centrifugation of the wastewater sample, the pellet is distributed along the side of the centrifuge tube facing the direction of the centrifugal force, but typically is not visible to the naked eye. Additionally, it takes time to dissolve the pellet in TRIzol, and premature decanting may leave residual RNA unrecovered. Therefore, untrained users often resuspend the pellet incompletely, resulting in the loss of valuable RNA. To aid in visualizing the pellet, we added safranin at 0.2% final concentration immediately before centrifugation. When safranin is added, a pale pink pellet is easily visible. Safranin staining increased yield and did not interfere with downstream processing (Fig 5; two-way ANOVA with Bonferroni correction: F = 18.87, df = 1, p = 0.0007). The video uploaded as (S1 Video) shows how long it takes to dissolve the pellet in TRIzol. This strategy of adding safranin is particularly useful for training purposes.

**Fig 6:**
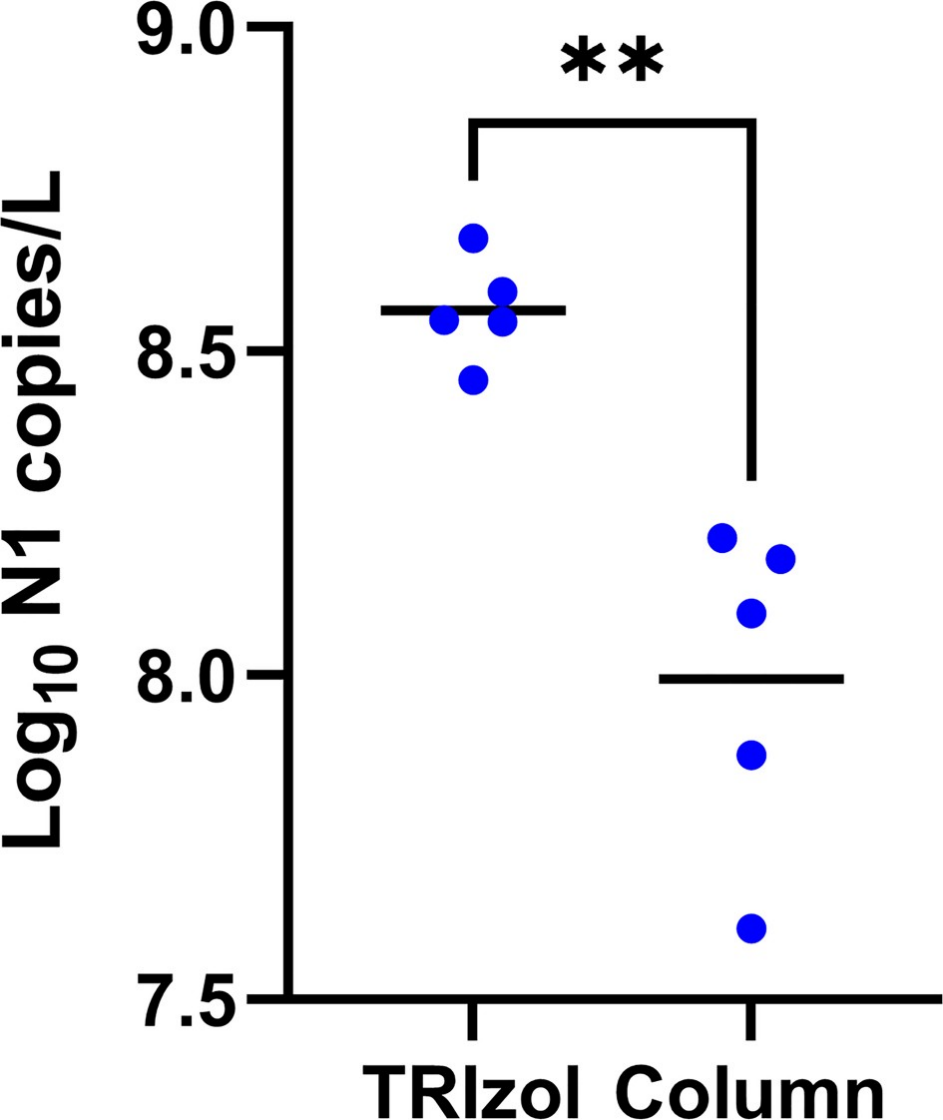
Effect of TRIzol extraction: Copy number yield for the N1 target for 24-hr composite wastewater samples obtained from 5 wastewater treatment plants. Each point in the mean of 2 technical replicate measurements from a 24-hour composite sample. Horizontal black lines show means. See Fig 2 legend for explanation of pairwise comparisons.

To explore the cheapest alternatives of extracting RNA from wastewater samples we compared a widely used column based QIAamp Viral RNA Mini kit (Qiagen Inc.) with TRIzol reagent (ThermoFisher Inc.). Our results showed that TRIzol facilitates significantly better RNA recovery than the kit at a fraction of the cost (Fig 6; paired t-test: F = 5.495, df = 4, P = 0.005). We note that we also found phenol-chloroform extraction to be less consistent than TRIzol on saliva samples so while phenol-chloroform is even cheaper, we advise against its use in this protocol. TRIzol was therefore chosen as the organic extraction method to compare with column approaches. If the intention is to sequence RNA obtained from wastewater samples, TRIzol extraction produces a cleaner RNA sample with less contaminating DNA from non-SARS-CoV-2 genomes. As a caveat, because TRIzol requires the careful extraction of an aqueous layer from a multilayered solution, TRIzol extraction requires training and is best performed by experienced users.

**Fig 7:**
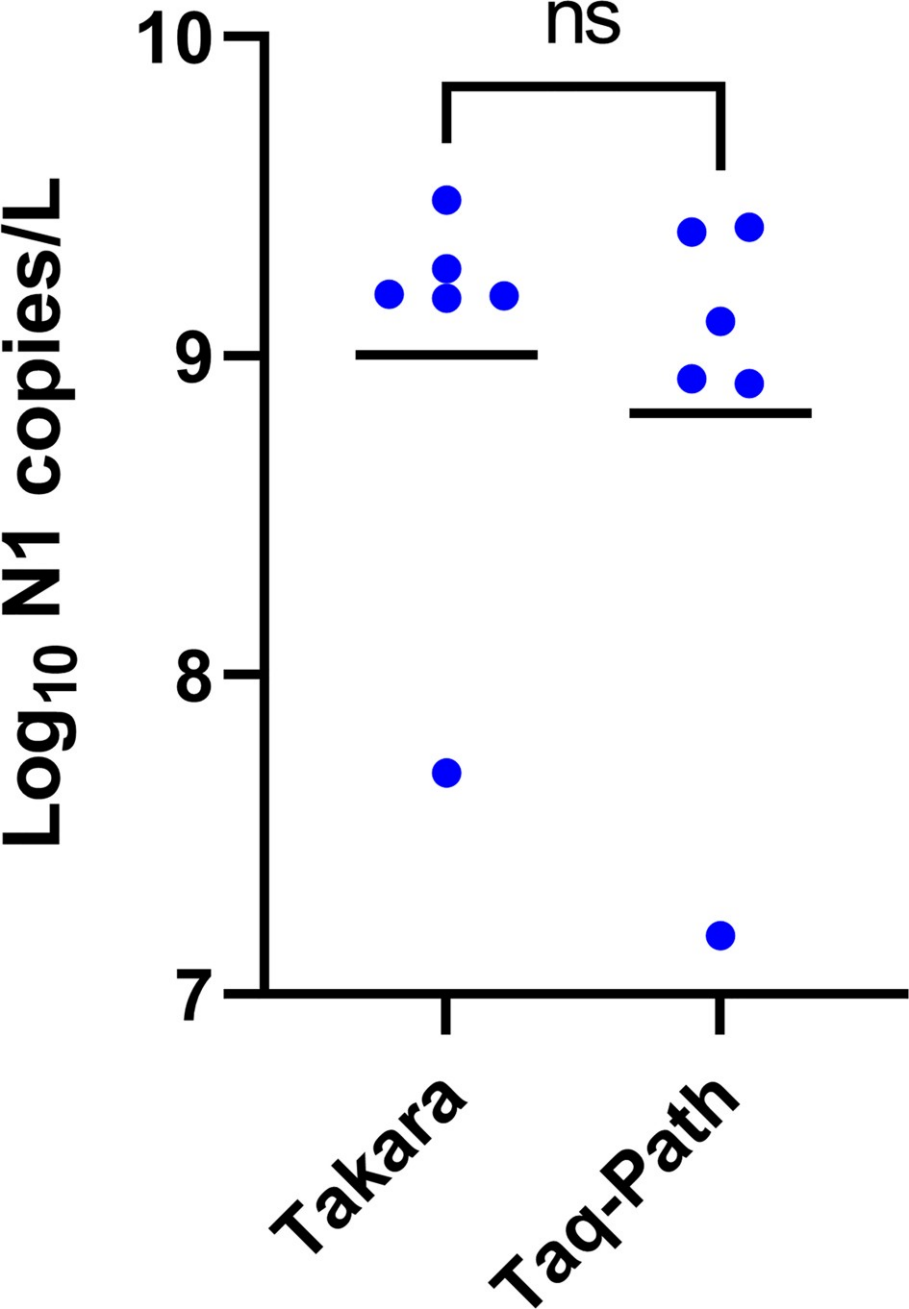
Effect of different RT-qPCR enzymes: Copy number yield for the N1 target for 24-hr composite wastewater samples obtained from 6 wastewater treatment plants (WWTP). RT-qPCR assays performed with the TaqPath 1-Step RT-qPCR enzyme recommended by the CDC [20] are compared to RT-qPCR assays performed with One Step PrimeScript III enzyme. Each point in the mean of 2 technical replicate measurements from a 24-hour composite sample. Horizontal black lines show means. See Fig 2 legend for explanation of pairwise comparisons.

In addition to comparing RNA isolation methods, we evaluated the performance of different enzymes, including the TaqPath 1-Step RT-qPCR enzyme (ThermoFisher Inc.) and the One Step PrimeScript III enzyme (Takara Bio USA Inc.) Our results indicated that the two enzymes performed equally well (Fig 7; paired t-test: t = 1.741, df = 5, p = ns). As the One Step PrimeScript III enzyme was significantly cheaper than the TaqPath enzyme, we chose the PrimeScript III enzyme for the final protocol.

The need to adapt wastewater surveillance detection programs to include variant detection requires deep sequencing of cDNA generated from the wastewater RNA. Our preliminary results have shown that RNA extracted with our PEG/TRIzol protocol can be sequenced using both traditional Sanger sequencing and NGS technology with no reduction in sequence quality.

We used both the Swift Normalase^®^ Amplicon Panel (SNAP) SARS-CoV-2 Panel kit as well as the Qiagen QIAseq® SARS-CoV-2 Primer Panel and QIAseq FX DNA Library kit and have obtained SARS-CoV-2 sequences from several of our wastewater treatment plants. Known and novel variants were identified. We continue to optimize and improve our library preparation methods to increase both length of coverage and depth of coverage for our NYC samples. In addition, we are developing real-time assays for the identification and quantification of additional viruses that circulate among our New York communities including Influenza.

## Supporting information

S1 File Step-by-step protocol, also available on protocols.io. (PDF)

S1 Video Showing the resuspension of the pellet with safranin. (M4V)
