# Supplementary material for: Protocol for safe, affordable, and reproducible isolation and quantitation of SARS-CoV-2 RNA from wastewater
Source: PLoS One. 2021 Sep 23;16(9):e0257454. doi: 10.1371/journal.pone.0257454 (PMC8459947; doi:10.1371/journal.pone.0257454)

Protocol for Safe, Affordable, and Reproducible Isolation of SARS-CoV-2 RNA from Wastewater V.2

Version 2

Aug 05, 2021

Monica Trujillo[1], Kristen Cheung[2], Anna Gao[2], Irene Hoxie[2,3], Sherin Kannoly[2], Kaung Myat San[2], Davida S. Smyth[4], John Dennehy[2,3]

[1]Department of Biology, Queensborough Community College, City University of New York, New York;

[2]Biology Department, Queens College, City University of New York, New York;

[3]Biology Doctoral Program, The Graduate Center, The City University of New York, New York;

[4]Department of Natural Sciences and Mathematics, Eugene Lang College of Liberal Arts at The New School, New York

2 *Works for me* Share

dx.doi.org/10.17504/protocols.io.bwvmpe46

Davida Smyth

ABSTRACT

The following protocol describes our workflow for processing wastewater with the goal of detecting the genetic signal of SARS-CoV-2. The steps include pasteurization, virus concentration, RNA extraction, and quantification by RT-qPCR. We include auxiliary steps that provide new users with tools and strategies that will help troubleshoot key steps in the process. This protocol is one of the safest, cheapest, and most reproducible approaches for the detection of SARS-CoV-2 RNA in wastewater. Furthermore, the RNA obtained using this protocol can be sequenced both using a targeted approach sequencing specific regions or the whole genome. The protocol was adopted by the NYC Department of Environmental Protection in August 2020 to support their efforts in monitoring SARS-CoV-2 prevalence in wastewater in all five boroughs of the city. Owing to the pasteurization step, it is safe for use in a BSL1+facility. This step does not reduce the genetic signal of the virus while making the protocol safe for the personnel involved. This protocol could be used to isolate a variety of other clinically relevant viruses from wastewater and serve as a foundation of a wastewater surveillance strategy for monitoring community spread of known and emerging viral pathogens.

DOI

dx.doi.org/10.17504/protocols.io.bwvmpe46

PROTOCOL CITATION

Monica Trujillo, Kristen Cheung, Anna Gao, Irene Hoxie, Sherin Kannoly, Kaung Myat San, Davida S. Smyth, John Dennehy 2021. Protocol for Safe, Affordable, and Reproducible Isolation of SARS-CoV-2 RNA from Wastewater. **protocols.io**
https://dx.doi.org/10.17504/protocols.io.bwvmpe46
Version created by Davida Smyth

KEYWORDS

RNA, Wastewater, Viral Concentration, Variants, RT-qPCR, PEG, TRIzol, Wastewater-based epidemiology

LICENSE

This is an open access protocol distributed under the terms of the Creative Commons Attribution License, which permits unrestricted use, distribution, and reproduction in any medium, provided the original author and source are credited

CREATED

Jul 25, 2021

LAST MODIFIED

Aug 05, 2021

OWNERSHIP HISTORY

Aug 05, 2021 Davida Smyth

PROTOCOL INTEGER ID

51853

GUIDELINES

- Use RNase-free materials throughout.
- Filter tips are required.
- We recommend using separate sets of pipettes where possible throughout the different stages of the protocol. Using the same pipettes for RNA isolation and RT-qPCR could result in cross-contamination.
- Method blanks can give positive signal if not enough care is taken to avoid cross-contamination. It is recommended to wipe with a diluted bleach solution the pipettes at the end of the day. Oak Ridge tubes must be left on a 20% bleach solution overnight, after rinsing them extensively they can be incubated at a 55 C incubator overnight or autoclaved. Care must be taken not to open the seal of the RT-PCR plates containing amplified N1 gene fragment, they should be disposed unopened.

MATERIALS TEXT

Equipment and Consumables

- –80 °C freezer to store water samples
- –20 °C freezer to store detection reagents
- 4 °C refrigerator storage of samples and reagents
- P10, P100 or P200, and P1000 pipettes. It is strongly recommended to use different sets of pipettes for the extraction of RNA and RT-qPCR.
- Centrifuge (up to 12,000 x g) and rotor for 50 mL tubes
- Water bath
- Polypropylene 50 mL Oak Ridge Tubes (ThermoFisher™Cat. No. 3139-0030)
- Corning™ 50 mL tubes (Fisher Scientific Cat. No. 430290)
- Filter tips to fit all pipette sizes. Only filtered tips should be used for each step in the protocol.
- 150 mL Vacuum Filter/Storage Bottle System, 0.22 µm Pore 13.6cm² CA Membrane (Corning™ 430769)
- Alternatively, 0.22 µm syringe filters have been used (Filters: MilliporeSigma™ Cat. No. SLGP033RB; Syringes: Becton Dickinson™ Cat. No. 309654)
- Pasteur pipettes (Fisher Scientific™ Cat. No. 22-183632)
- 1.5mL microcentrifuge tubes (USA Scientific Cat. No. 1615-5510)
- 0.2 mL PCR tubes (Eppendorf™0030124847)
- Applied Biosystems™ MicroAmp™ Fast Optic 48-Well Reaction Plate (ThermoFisher Scientific Cat. No. 43-758-16).

Solutions/Reagents

- Polyethylene glycol 8000 (Fisher Scientific™ Cat. No. BP233-1)
- Sodium chloride (Fisher Scientific™Cat. No. BP358-10)
- TE buffer (Fisher Scientific Cat. No. BP2473500).
- TRIzol™ Reagent (Invitrogen™ Cat. No. 15596018)
- Chloroform (Fisher Scientific™ Cat. No. BP1145-1)
- Nuclease-free DEPC-treated water (Cat. No. AM9906) (10 x 50 mL)
- Ethanol, Absolute (200 Proof), Molecular Biology Grade (Fisher Scientific™ A4094)
- 2% Safranin Solution (Sigma-Aldrich Cat. No. 1092170500)
- Isopropanol (Fisher Scientific™ BP26181)
- Takara One Step PrimeScript III RT-PCR Kit (2x) (Takara™ RR064B)
- ROX Reference Dye (Invitrogen™ LS12223012)
- 2019-nCoV RUO N1 primers/probe (IDT) (Cat. No.10006713) (750 µL; 500 reactions)
- Custom BCoV qPCR assay (primers/probe)(IDT)
- Twist RNA (Cat. No. SKU# 102019)
- BCoV Standard gBlocks® Gene Fragment
- Calf-Guard® Bovine Rota Coronavirus vaccine (Zoetis, Cat. No. 540463)

SAFETY WARNINGS

SARS-CoV-2 is designated a BSL3 pathogen. Wastewater samples should be pasteurized prior to processing for RNA isolation. Appropriate PPE should be donned throughout processing the samples.

Handle chloroform with care. Consult the SDS before use.

Day 1 - Pasteurization and Solids removal 1d

1 **Sample handling** 1d

The samples must be kept at 4°C at all times after collection. Keep wastewater sample containers closed throughout the entire procedure except where noted.

2 **Pasteurization**

In our protocol, samples are pasteurized the day they are received in the lab to minimize exposure to pathogens. The pasteruized samples are subsequently centrifuged to remove bulk solids, filtered to remove particulates, spiked with BCoV control virus, and placed overnight in PEG/NaCl solution for virion precipitation. Virion concentration, RNA extraction, and RT-qPCR is performed on day two. We recommend performing all activities in a biological safety cabinet to minimize exposure/contamination. SARS-CoV-2 appears to be stable and able to persist in laboratory surfaces and instruments so dedicated cleaning/decontamination procedures are indicated.

1. The wastewater samples in their original containers (~240 ml) are placed on a dedicated, preheated 60°C water bath. Once the wastewater container is in the water bath one of the samples is briefly opened to introduce a temperature probe and closed immediately. Wastewater samples are pasteurized for 60 minutes after the internal temperature of the wastewater samples has reached 60 °C. In our experience, it took between 30-60 minutes for the samples to reach 60°C.
2. Prepare a method blank by adding 40 mL of deionized water into a separate, labeled 50 mL conical tube.
3. After pasteurization, remove containers from water bath, wipe away any remaining liquid outside each container and aliquot 40 ml into 50 mL conical tubes.
4. For standard detection, remove 40 mL of the pasteurized sample and continue with solid separation.

3 Control Spike-In and Solids Separation

1d

1. Thaw a vial of Calf-Guard® Bovine Rota Coronavirus vaccine (Zoetis Cat. No. 11712) on ice. Calf-Guard contains an attenuated bovine coronavirus (hereafter BCoV). To prepare a BCoV control spike-in, dissolve the vaccine in 1 mL of TE buffer (Fisher Scientific Cat. No. BP2473500). Divide the resuspended BCoV control spike-in into 10 separate 100 µL aliquots and store them at -20 °C. These are single-use aliquots. Dilute BCoV 1:10 in nuclease-free DEPC-treated water (Fisher Scientific Cat. No. AM9906).
2. Add 40 µl of the 1:10 BCoV control spike-in to each conical tube. Load centrifuge tubes in the rotor. Centrifuge the samples at 5000 x g for 10 min at 4 °C with the brake set at 0. We used a Sorvall RC5B centrifuge with an SS34 rotor.

Note: The paper that accompanies this protocol includes data that demonstrates the positive effect of pasteurization and of storing the samples in PEG/NaCl at 4 °C for at least 24 hours. The protocol includes a control for determining recovery efficiency. This is achieved by spiking the wastewater samples with a known amount of a bovine coronavirus. The protocol was initially developed using Phi6, but due to the rapid degradation of Phi6 in wastewater, we switched to bovine coronavirus.

4 Supernatant Filtration

1. Without disturbing the pellet, carefully transfer 40 mL of each sample supernatant to a 150 mL Vacuum Filter/Storage Bottle System (Corning™ Cat. No. 430769) or load a 50 mL syringe (Becton Dickinson™ Cat. No. 309654) that has a 0.22 µm filter (MilliporeSigma™ Cat. No. SLGP033RB) attached to it.
2. Attach a disposable vacuum filter system to the laboratory stationary vacuum line or to a manifold connected to an electric vacuum pump. Turn on the vacuum line and filter until all liquid has passed through the filter. If using a syringe filter, pass the wastewater through the filter manually.

3. Transfer filtrate to a 50 mL polypropylene Oak Ridge tube (ThermoFisher™ Cat. No. 3139-0030).

Note: Tubes and syringes can be reused if bleach-sterilized and autoclaved.

5 **Virus Concentration** 1d

1. Using an analytical balance, weigh out 0.9 g sodium chloride (NaCl; Fisher Scientific™ BP358-10) and 4.0 g PEG 8000 (Fisher Scientific™ BP233-1) and add to each sample tube.
2. Mix by inversion until the PEG/NaCl solids have dissolved and the mix is translucent.
3. Store the samples overnight in a refrigerator at 4 °C.

Note: Longer incubation times do not reduce the precipitation of the virus. In fact, a 48 hr incubation increased the yield of the genomic signal of the virus.

Day 2 1d

6 **Centrifugation** 1d

1. Remove tubes from the 4 °C refrigerator and add 100 µl of a 2% safranin solution (Sigma-Aldrich Cat. No. 1092170500) to the tubes. Invert the tubes several times to mix well.
2. Prior to centrifugation, mark the tube where the pellet is expected to precipitate. This will help determine pellet location when examining later.
3. Centrifuge the tubes at 12,000 x g for 120 minutes at 4 °C with the brake set at 0. If no safranin is added, the pellet may be invisible. With added safranin, the pellet appears pale pink.
4. Remove the majority of supernatant by decanting. Pour supernatant carefully from the side of the tube opposite to the pellet. Collect the supernatant in a liquids container as biohazardous waste to be autoclaved.
5. Centrifuge the tubes again at 12,000 x g for 5 minutes at 4 °C.
6. Remove any remaining liquid with a sterile glass Pasteur pipette (Fisher Scientific™ Cat. No. 22-183632).

Note: When using Pasteur pipettes, care must be taken to decontaminate the rubber bulbs by cleaning with 10% bleach and leaving overnight.

7 **RNA Extraction**

TRIzol protocol

Phase Separation

1. Resuspend the pellet with 1.5 mL of TRIzol (Invitrogen™ Cat. No. 15596018) by shaking the tube vigorously for at least 45 seconds*.
2. Let the tube rest at room temperature for 5 minutes.
3. Split the 1.5 mL of TRIzol resuspension into 2 equivalent amounts and transfer to 1.5mL microcentrifuge tubes (USA Scientific 1615-5510).
4. Add 150 µl of chloroform (Fisher Scientific™ Cat. No. BP1145-1) to TRIzol resuspension to each of the tubes. (chloroform:TRIzol = 1:5 ratio). Invert the tube several times to mix.
5. Centrifuge the sample at 12,000 g for 15 minutes at 4 °C.

6. Carefully remove the aqueous phase (top layer) from each tube and pool it into one tube. For best results, try not to aspirate any of the bottom layer. It is better to not recover the whole top layer than accidentally aspirate the bottom layer.

* It is crucial for the success of the protocol to completely dissolve the pellet before extracting RNA. We have developed a visualization step using the staining agent safranin that is strongly recommended with this protocol.

Note: Handle chloroform with care and use a fume hood/biological safety cabinet to avoid inhaling fumes. Consult the SDS before use.

RNA Extraction

1. Add 600 µl of isopropanol (Fisher Scientific™ Cat. No. BP26181) into the aqueous phase (isopropanol: aqueous phase = 1:1 ratio), and invert the tube several times to mix.
2. Incubate at room temperature for 15 minutes.
3. Centrifuge at 12,000 g for 10 minutes at 4 °C. A pink pellet can be seen at the bottom or on the side of the tube.
4. Decant and discard the supernatant.

RNA Wash and Elution

1. Add 1 mL of 75% ethanol (Fisher Scientific™ Cat. No. A4094) (100% ethanol:nuclease-free water = 3:1) to wash the pellet.
2. Centrifuge 12,000 g for 3 minutes at 4 °C. Discard supernatant.
3. Repeat wash once again with 1 mL of freshly made 75 % ethanol.
4. Discard supernatant, air dry for 5-10 minutes (do not over-dry the pellet).
5. Resuspend the pellet in 30 µl of nuclease-free water , let it sit at room temperature for 5 minutes before any downstream steps.

Day 3 1d

8 **RT-qPCR for N1 Target**

Master Mix

1. To prepare a master mix, use the table below. N = number of samples (note: if N > 10, add N + 3 for the volume for each step).
2. Obtain One Step PrimeScript™ RT-PCR master mix (Takara™ Cat. No. RR064B) and ROX Reference Dye (Invitrogen™ Cat. No. LS12223012) from the -20 °C freezer, and keep on ice.
3. Aliquot the appropriate amount of the One Step PrimeScript™ RT-PCR master mix and ROX Reference Dye to a 1.5mL microcentrifuge tube.
4. Add the calculated amount of N1 primer/probe mix (IDT Cat. No. 10006713) then add nuclease-free water.
5. Mix the One Step PrimeScript™ RT-PCR master mix, ROX Reference Dye, nuclease-free water, and N1 primers/probe by pipetting up and down 15 times and return to ice.

6. Using a multichannel pipette, place 15 µl of master mix into each reaction well of Applied Biosystems™ MicroAmp™ Fast Optic 48-Well Reaction Plate (ThermoFisher Scientific Cat. No. 43-758-16).
7. Continue adding 5 µl of each sample to each reaction well.

Sequences of the primers and probe used :
2019-nCoV_N1 Forward Primer: GAC CCC AAA ATC AGC GAA AT
2019-nCoV_N1 Reverse Primer : TCT GGT TAC TGC CAG TTG AAT CTG
2019-nCoV_N1 Probe: FAM-ACC CCG CAT /ZEN/ TAC GTT TGG TGG ACC-3IABkFQ

A	B	C
A	B	C
Step #	Reagent	Volume of reagent added per reaction
1	One Step PrimeScript™ RT-PCR master mix	N x 10 µl
2	ROX Reference Dye	N x 0.4 µl
3	N1 probe/primers	N x 1.5 µl
4	RNase free water	N x 3.1 µl
	Total Volume	N x 15 µl

Table 1. Plate set up RT-qPCR for N1 Target

Standard Curve with Twist

1. Remove an aliquot of the Twist Synthetic SARS-CoV-2 RNA Control 1 (Twist Biosciences Cat. No. MT007544.1) from the –80°C freezer and thaw on ice.
2. Obtain a strip of 0.2 mL PCR tubes (Eppendorf™0030124847) and label the tubes “STD 2” - “STD 6.”
3. Place the tubes on ice.
4. Follow the table below to obtain the standards.
5. Use a new tip for each transfer and mix by pipetting up and down 15 times.
6. Close the tubes and keep the standards on the cold PCR tube rack as they are made.
7. Starting with the STD 6, add 5 µl of each dilution to the proper reaction well of an Applied Biosystems™MicroAmp™ Fast Optical 48-Well Reaction Plate (ThermoFisher Scientific Cat. No: 4375816)
8. Seal the 48-Well Reaction Plate with Adhesive PCR Plate Seal (ThermoFisher Scientific Cat. No: AB0558) using a plate roller.
9. Centrifuge 48-Well Reaction Plate for 5 minutes at 2097 x g to ensure that there are no bubbles in any reaction wells.
10. Place the well into an Applied Biosystems StepOnePlus instrument.
11. Start the reaction according to the following cycling parameters.

A	B	C	D
**Standard**	**Vol of nuclease-free water (µL)**	**Stock to use in serial dilution**	**Vol of stock (µL)**
**STD 1**	**36**	stock, frozen in aliquots upon receipt	in stored aliquot tube 4
**STD 2**	**36**	**STD 1**	**4**
**STD 3**	**36**	**STD 2**	**4**
**STD 4**	**36**	**STD 3**	**4**
**STD 5**	**36**	**STD 4**	**4**
**STD 6**	**20**	**STD 5**	**20**
**STD 7**	**32**	**STD 6**	**8**

Table 2. Dilution series for Twist Standard Curve

A	B	C	D
	Temp ˚C	Time	Cycles
Stage 1	25	2 min	1
Stage 2	50	15 min	1
Stage 3	95	2 min	1
Denature	95	3 sec	45
Anneal and Extension	55	30 sec	

Table 3: Cycling conditions for N1 fragment amplification

RT-qPCR for BCoV Targets
Master Mix

1. For a stock of master mix, use the table below. N = number of samples (note: if N > 10, add N + 3 for the amount of volume for each step).
2. Obtain One Step PrimeScript™ RT-PCR master mix from the -20°C refrigerator, and keep on ice.
3. Aliquot the appropriate amount of One Step PrimeScript™ RT-PCR master mix to a 1.5 mL microcentrifuge tube.
4. Add the calculated amount of BCoV primer/probe mix then add nuclease-free water.
5. Mix the One Step PrimeScript™ RT-PCR master mix, nuclease-free water, and BCoV probe/primers by pipetting up and down 15 times and return to the PCR tube rack.
6. Using the multichannel pipette, place 15 µl of master mix into each reaction well.
7. Add 5µl nuclease-free water to well A1 as a negative control.
8. Continue adding 5 µl of each sample to the proper reaction well

BCoV Forward Primer: CTGGAAGTTGGTGGAGTT
BCoV Reverse Primer: ATTATCGGCCTAACATACATC
BCoV Probe: FAM-CCTTCATATCTATACACATCAAGTTGTT-3IABkFQ

A	B	C
A	B	C
Step #	Reagent	Volume of reagent added per reaction
1	One Step PrimeScript™ RT-PCR master mix	N x 10 µl
2	ROX Reference Dye	N x 0.4 µl
3	BCoV probe/primers	N x 1.5 µl
4	RNase free water	N x 3.1 µl
	Total volume	N x 15 µl

Table 4. Plate set up for RT-qPCR for BCoV probe

Standard Curve

1. Remove an aliquot of the custom-made BCoV Standard gBlocks® Gene Fragments (IDT Inc.) from the –20 °C freezer and thaw on ice.
2. Obtain a strip of PCR tubes and label the tubes "STD 2" - "STD 6."
3. Place the tubes on the ice.
4. Follow the table below to obtain the standards.
5. Use a new pipet tip for each transfer and mix by pipetting up and down 15 times.
6. Close the tubes and keep the standards on ice while preparing all dilutions.
7. Starting with the STD 6, add 5 µl of each dilution to each reaction well.
8. Seal theFast Optical 48-Well Reaction Plate with Adhesive PCR Plate Seal using a plate roller.
9. Centrifuge for 5 minutes at 2097 x g and ensure that there are no bubbles in any reaction wells.
10. Place the well into the StepOnePlus instrument.
11. Start the reaction according to the following cycling parameters

Sequence of the BCoV Standard gBlock gene fragment:
GTATCAGGTTGTTTATTAGAACTGGAAGTTGGTGGAGTTTCAACCCAGAAACAAACAACTTGATGTGTATAGATATGAAGGGAAGGATGTATGTTAGGCCGATAATTGAGGACTACCATACCCTTA

A	B	C	D
**Standard**	**Vol of nuclease-free water (µL)**	**Stock to use in serial dilution**	**Vol of stock (µL)**
**STD 1**	**18**	stock as shipped, frozen in aliquots upon receipt	will already be in stored aliquot tube 5
**STD 2**	**18**	**STD 1**	**2**
**STD 3**	**18**	**STD 2**	**2**
**STD 4**	**18**	**STD 3**	**2**
**STD 5**	**18**	**STD 4**	**2**
**STD 6**	**18**	**STD 5**	**2**
**STD 7**	**18**	**STD 6**	**2**
**STD 8**	**18**	**STD 7**	**2**
**STD 9**	**18**	**STD 8**	**2**
**STD 10**	**18**	**STD 9**	**2**
**STD 11**	**18**	**STD 11**	**2**

Table 5: Dilution series of the BCoV Standard gBlock gene fragment used for the standard curve.

A	B	C	D
	Temp ˚C	Time	Cycles
Stage 1	25	2 min	1
Stage 2	50	10 min	1
Denature	95	2 min	1
Denature	95	15 sec	45
Anneal	60	30 sec	
Extend	60	1 min	

Table 6: Cycling conditions for BCoV gBlock gene fragment amplification

9 **Data analysis and interpreting the standard curve using the StepOnePlus™**

1. View the amplification plot, and modify as needed. Use the default baseline and threshold values.
2. In the well table or results table, view the Ct values for each well and for each replicate group.
3. Generate slope, amplification efficiency, R^2 values from the standard curve.
4. Adhere to Minimum Information for Publication (MIQE) standards for publishing RT-qPCR data.

Sample output file: **sample for RT-qPCR.xls**

Assay Performance Standards

- Ct < 40
- Ct values of replicates should vary by no more than 0.2 standard deviation units

- Sensitivity--accurate detection of 20 copies
- Amplification efficiency--90–110%
- Linear dynamic range--minimum of six orders of magnitude. Detection of a synthetic template standard curve from 20 to 20 million copies
- Standard curve slope = -3.32
- Standard curve coefficient of correlation R^2 > 0.97

Using a pooled standard curve for data analysis

10

- Using a pooled standard curve is very useful when quantifying for extended periods of time. Using the same synthetic template standard we stored over 40 standard curves and averaged them. The pooled average standard curve was used to calculate copies/L. However, a standard curve was run every time plates were run. The corresponding standard curve was compared with the pooled average curve. If the amplification efficiency was not between 90-110%, the standard curve coefficient of correlation (R^2) was less than 0.97 and/or the slope differed by more than 10% compared to the slope from the pooled average curve, the samples were re-run.

Pooled average standard curve

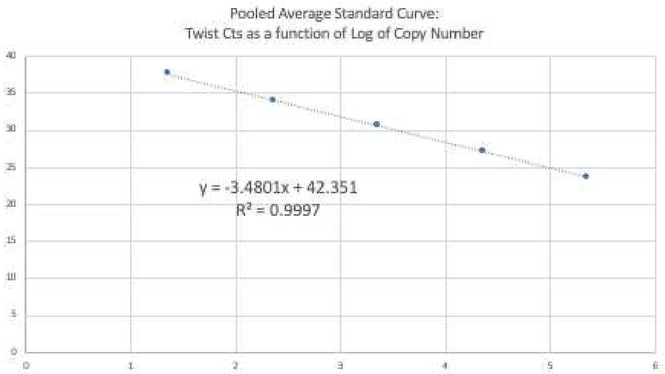

Supplement: S1 File — (PDF) [file pone.0257454.s001.pdf]
